# Stability of Radiomic Features against Variations in Lesion Segmentations Computed on Apparent Diffusion Coefficient Maps of Breast Lesions

**DOI:** 10.3390/diagnostics14131427

**Published:** 2024-07-03

**Authors:** Mona Pistel, Luise Brock, Frederik Bernd Laun, Ramona Erber, Elisabeth Weiland, Michael Uder, Evelyn Wenkel, Sabine Ohlmeyer, Sebastian Bickelhaupt

**Affiliations:** 1 Institute of Radiology, University Hospital Erlangen, Friedrich-Alexander-Universität Erlangen-Nürnberg (FAU), 91054 Erlangen, Germany; 2 Siemens Healthineers AG, 91052 Erlangen, Germany; 3 Institute of Pathology, Friedrich-Alexander-Universität Erlangen-Nürnberg (FAU), 91054 Erlangen, Germany; 4 MR Application Predevelopment, Siemens Healthineers AG, 91052 Erlangen, Germany; 5 Radiologie München, 80331 München, Germany

**Keywords:** radiomic stability, diffusion magnetic resonance imaging, breast lesions

## Abstract

Diffusion-weighted imaging (DWI) combined with radiomics can aid in the differentiation of breast lesions. Segmentation characteristics, however, might influence radiomic features. To evaluate feature stability, we implemented a standardized pipeline featuring shifts and shape variations of the underlying segmentations. A total of 103 patients were retrospectively included in this IRB-approved study after multiparametric diagnostic breast 3T MRI with a spin-echo diffusion-weighted sequence with echoplanar readout (b-values: 50, 750 and 1500 s/mm^2^). Lesion segmentations underwent shifts and shape variations, with >100 radiomic features extracted from apparent diffusion coefficient (ADC) maps for each variation. These features were then compared and ranked based on their stability, measured by the Overall Concordance Correlation Coefficient (OCCC) and Dynamic Range (DR). Results showed variation in feature robustness to segmentation changes. The most stable features, excluding shape-related features, were FO (Mean, Median, RootMeanSquared), GLDM (DependenceNonUniformity), GLRLM (RunLengthNonUniformity), and GLSZM (SizeZoneNonUniformity), which all had OCCC and DR > 0.95 for both shifting and resizing the segmentation. Perimeter, MajorAxisLength, MaximumDiameter, PixelSurface, MeshSurface, and MinorAxisLength were the most stable features in the Shape category with OCCC and DR > 0.95 for resizing. Considering the variability in radiomic feature stability against segmentation variations is relevant when interpreting radiomic analysis of breast DWI data.

## 1. Introduction

Personalized medicine is an aspired goal of modern oncology. In this context, the treatment of cancer is individually adapted to the patient and their respective tumor characteristics. Treatment pathways thus are increasingly influenced by analyses of tumor-specific comprehensive signatures obtained by tissue biopsy.

In addition to the fact that this procedure is invasive and therefore limited at least to a certain degree for repetitive longitudinal monitoring purposes, the information obtained by biopsy is only based on a small fraction of the tumor. For heterogeneous tumor tissue, a biopsy might be limited in providing holistic information [1,2,3]. Advancing non-invasive and at the same time holistic extraction of tumor signatures is therefore of great relevance in personalized medicine. Merging advanced imaging technology with sophisticated data analyses might support approaching this aim for personalized medicine. In this approach, a large number of radiological image data-derived features are extracted and combined by means of statistical models to predict biological and diagnostic relevant points [4]. Radiomics, one such approach, enables the capture of quantitative features that are potentially hidden from the human eye and therefore represent a promising tool in personalized medicine as a digital signature [5,6,7,8,9]. Even though a large number of features can be extracted with radiomics, it is advisable to acknowledge its limitations, especially with regards to the stability of the features. An important first step after feature extraction is therefore the selection of stable features, allowing for the identification of those sufficiently robust for clinical use [10]. The literature describes different procedures to test feature stability. Peerlings et al., for example, evaluated the stability of radiomic features in diffusion-weighted imaging (DWI) by means of a test–retest study [11]. Ramli et al., on the other hand, investigated feature stability in DWI by using different segmentations for the same lesion and subsequently comparing the features [12].

Here, DWI is of dedicated interest in quantitative oncologic imaging. Beyond relative signal intensity, it allows one to gain quantitative insight into tissue microstructure [13]. Mapping the random Brownian motion of water molecules in the tissue by using DWI can provide information about oncologically relevant changes in the tissue examined [14,15,16,17]. As an imaging sequence that does not need contrast agent application, it is further of high interest for the longitudinal, repetitive monitoring of oncologic patients aiming to support treatment decisions in patient care. DWI is increasingly incorporated in multiparametric breast MRI protocol, where it has been described to help in detecting and differentiating breast lesions non-invasively [14,15,18,19]. Together with radiomics, a non-invasive and at the same time holistic extraction and interpretation of breast tumor characteristics could thus be achieved and help to improve breast cancer pathways. To ensure the repeatability and reproducibility of radiomic models, extracted DWI breast features need to be tested for stability.

The aim of the following study was thus to test radiomic feature stability for breast DWI by implementing a standardized pipeline featuring shifts and shape variations of the underlying segmentations, mimicking variations in segmentations. Stability in this context refers to the resilience of the feature to these small discrepancies in segmentation.

## 2. Materials and Methods

### 2.1. Patients

A retrospective analysis of previously prospectively acquired breast MRI examinations was performed. Written informed consent had been obtained from all patients prior to inclusion in the study after the approval of the study protocol by the local ethics committee. All examinations were performed between March 2017 and January 2020. One hundred and twenty-five patients (*n* = 125), aged between 24.1 and 86.4 years, were included in the study. The breast MRI datasets used in the current evaluation had been previously analyzed with different objectives [18,20]. However, these previous studies were not radiomics-focused, nor did they analyze the stability of extracted image information. For convenience, we reiterate the description for patient characteristics and MRI acquisition here.

All patients included in this study fulfilled one of the following inclusion criteria for undergoing a breast MRI examination: participating in screening for women with a high risk of breast cancer, suspicious or inconclusive sonography or mammography, or a history of breast cancer with the indication for breast MRI as part of the follow-up care. Amongst the study population, forty-seven patients (*n* = 47) had more than one lesion. If one or more lesions had to be excluded from a patient with multiple lesions, the patient was still included in the study, if at least one of her lesions fulfilled the inclusion criteria.

Biopsy was performed for suspicious lesions detected in the breast MRI examination in order to obtain histopathology. Biopsy was performed by core-needle biopsy, vacuum biopsy, and/or surgery. Lesions without biopsy were assigned to the group of benign lesions if they presented as stable in the follow-up examinations for more than 12 months or if they were defined as BI-RADS 2 lesions in complementary/follow-up sonography. Benign lesion classification was performed by radiologists (S.O. and E.W. with over 5 and 17 years of experience in breast MRI, respectively).

### 2.2. MRI Examinations

All patients included in the study were examined using clinical routine 3 T MRI scanners (*n* = 108 patients using a MAGNETOM Skyra and *n* = 17 patients using a MAGNETOM Vida, both Siemens Healthineers AG, Erlangen, Germany) as previously described [17]. Patients were examined in the prone position using an 18-channel bilateral breast coil (Siemens Healthineers AG, Erlangen, Germany). A full standard diagnostic breast MRI protocol was acquired including an additive vendor-provided prototype DWI echo planar imaging sequence. The sequence parameters of the DWI sequence and of the used dynamic contrast-enhanced (DCE) sequence are stated in Table 1. Apparent diffusion coefficient (ADC) maps were calculated in Matlab 2020b (The MathWorks, Inc., Natick, MA, USA) based on the b50 = 50 s/mm^2^ and b750 = 750 s/mm^2^ images.

### 2.3. Image Analysis

All DWI data used for this evaluation were extracted from the clinical PACS (Picture Archiving and Communication System) and transferred to a research workstation. All slices containing a lesion were visually checked for sufficient image quality by M.P. (physicist with four years of experience in breast MRI). Data that did not pass this quality check were excluded from further analysis. In particular, lesions were excluded if one of the following criteria was met, as laid out in detail in the results part: intolerably large image distortion in the slice of the lesion, insufficient fat suppression, lesion distorted by biopsy, or because the lesion was not visible in the b = 1500 s/mm^2^ or b = 750 s/mm^2^ image or detailed lesion classification, as well as DCE images being missing.

For two lesions (one benign and one malignant), the b750 and b1500 images had to be co-registered to the b50 images for motion correction. Rigid image registration was performed with the Fijiyama plugin for ImageJ 1.53c (National Institutes of Health, Bethesda, MD, USA) [21].

**Figure 1:**
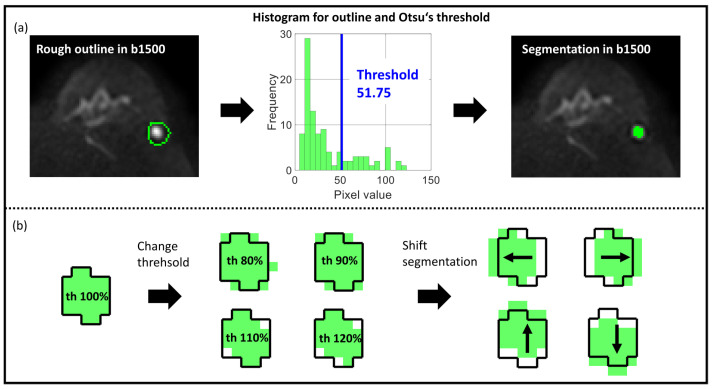
Otsu’s threshold method for segmentation. (**a**) Segmentation workflow with rough delineation in the b1500 image to simplify the thresholding process, Otsu’s threshold in the histogram, and final segmentation in the b1500 image. (**b**) Two variations of original segmentation (th = 100%): Resize by changing threshold (th = 80%, th = 90%, th = 110%, th = 120%) and shift of segmentation by one pixel (shift x1, shift -x1, shift y1, shift -y1). Bright green area in (**b**) indicates the segmentation mask, the dark lined area the lesion as segmented with th = 100%, the arrows show the direction in which the segmentation is shifted.

Figure 1a depicts the workflow. First, the b1500 images were opened in Matlab 2020b and used to roughly delineate the lesion in the slice with the largest lesion area. If a lesion was not clearly visible in the b1500 image, the b750 image was used for this step. Lesion location in Matlab was compared with DCE images (third subtraction after 3 min) in the Medical Imaging Interaction Toolkit (MITK, DKFZ, Heidelberg, Germany). For lesions without contrast enhancement, lesion location was compared with the apparent diffusion coefficient (ADC) map. Second, Otsu’s threshold was determined for the gray level histogram of the delineated 2D region by separating the histogram into two classes and selecting the threshold by the so-called discriminant criterion using in-house software, developed in Matlab [22]. This criterion maximizes the variance between the two gray level classes. We decided to use Otsu’s threshold because of its suitability for handling black-and-white contrasts and because it could be used easily to adjust the segmentation size mimicking radiologists going for somewhat smaller or larger segmentations based on the perceived signal intensity. Third, lesions were segmented by including all voxel values above the determined threshold and excluding the voxels equal or below the given threshold value.

To verify the validity of the threshold-based segmentations, they were compared to previously obtained manual segmentations (in [17], segmented by the physicist M.P. with 3 years of experience, trained by the board-certified radiologist S.O.).

Special attention was paid to the following items:

- Most peripheral lesion parts should be excluded from the segmentation;
- Necrotic and fatty regions should be excluded;
- In cases where biopsy was performed before MR imaging took place, the biopsy-affected region should be excluded from the segmentation.

If the Otsu-based method provided a non-acceptable segmentation based on these criteria, the lesion was again delineated in the b1500 (or b750) image and a new Otsu’s threshold was calculated and used for segmentation.

All final segmentations were subjected to the following variations (see Figure 1b):

- Resizing: Change in the segmentation threshold to 80%, 90%, 110%, and 120%
- Shift: Shift of segmentation in four directions by one voxel (left, right, ventral, and dorsal).

Thus, together with the initial 100% segmentation, nine different segmentations for every lesion were available and used for further analysis.

### 2.4. Radiomic Feature Extraction

Radiomic features were extracted from the ADC maps for all segmentations and lesions. ADC fit outliers outside the range [0, 3.5] μm^2^/ms were excluded. The choice of the upper limit was based on the expected ADC value of 3.25 μm^2^/ms for free water [23,24]. PyRadiomics (Version 3.0.1) was used to extract the features [25]. The recommendations of the Image Biomarker Standardisation Initiative (IBSI) were followed [26]. Settings for feature extraction in the current study are listed in the Supplementary Materials (see Table S1). In total, 102 features from seven different feature classes (Shape, First Order (FO), Gray Level Co-occurrence Matrix (GLCM), Gray Level Run Length Matrix (GLRLM), Gray Level Dependence Matrix (GLDM), Gray Level Size Zone Matrix (GLSZM), and Neighboring Gray Tone Difference Matrix (NGTDM)) were extracted. Details about the feature classes can be found in the Supplementary Materials (see section Extracted features, Figures S1–S3).

### 2.5. Stability Score

A stability score for our analysis was determined, which includes the Overall Concordance Correlation Coefficient (OCCC) and the Dynamic Range (DR) (see Table 2 for score details). It is based on two different variations of the segmentation, namely changing the threshold of the Otsu method (resizing) and shifting the segmentation (shifting). Only resizing has an effect on the shape of the segmentation. For Shape features, therefore, only the five segmentations from the varying Otsu’s thresholds were considered per lesion. Shape features were not included in the resulting stability plot comprising the scores from shifting and resizing but were discussed separately.

The OCCC was calculated to test the consistency of extracted features between different segmentations. According to Lin et al. and Barnhart et al., the OCCC evaluates the agreement of continuous measured values and is calculated as follows [27,28]:

(1) $$\mathrm{OCCC}\;=\frac{2\sum_{j=1}^{J-1}\sum_{k=j+1}^{J}\sigma_{jk}}{\left(J-1\right)\sum_{j=1}^{J}\sigma_{j}^{2}+\sum_{j=1}^{J-1}\sum_{k=j+1}^{J}{\left(\mu_{j}-\mu_{k}\right)}^{2}},$$

with *μ* being the mean of all values per feature, *σ* being the corresponding variances and covariances, *k* and *j* being the segmentations to be compared, and *J* being the total number of segmentations per lesion.

OCCC can be seen as a generalization of the Concordance Correlation Coefficients (CCCs), which can determine correspondences only between two measured values [28]. OCCC values are in the range [−1; 1], where an OCCC of 1 means a complete agreement of the compared measured values and a value of −1 means a complete inverse match [28]. In this work, the OCCC was calculated using the epiR package for R [29].

In addition to feature consistency, the DR was determined to obtain an impression of features of inter-patient variability. DR is a measure that combines the natural range of the data with its reproducibility. Adapted from Balagurunathan et al., DR is defined as the inverse of the average difference of the measured values divided by their total range [30]:

(2) $$\mathrm{DR}=1-\frac{1}{n}\sum_{i=1}^{n}\frac{\left|f\;(\mathrm{Test}(i))-f\;(\mathrm{Retest}(i))\right|}{\mathrm{Max}-\mathrm{Min}},$$

where *i* refers to an individual lesion ranging from 1 to *n*. *f(*Test*(i))* describes feature *f* of lesion *i* extracted from the original segmentation Test*(i)*. *f(*Retest*(i)),* on the other hand, describes the feature of lesion *i* extracted from the varied segmentation Retest*(i)*. In this work, *Max* and *Min* refer to the largest and smallest feature values of the original segmentation amongst all lesions of all patients. DR is calculated for all four retest segmentations and averaged afterwards. DR is defined in the range [0; 1]. Larger DRs imply a large natural range compared to feature reproducibility. A stable feature therefore tends to have large DR values.

For each feature (excluding Shape features), the DR and OCCC score was determined twice, once for resizing and once for shifting. The classification of OCCC and DR into five points is based on reported studies and their considerations for feature stability, which have shown to effectively balance sensitivity and specificity in feature rankings, providing a robust framework for comparative analysis [30,31,32,33]. The stability score was defined as the sum of the scores of OCCC and DR (c.f. Table 2).

In total, a feature can score a maximum of four points per statistical measure, meaning a maximum of 8 = 4 + 4 points for both measures. In order to rank the features according to their stability, points from both categories (shifting and resizing) were plotted and the distance to the maximum achievable point (8, 8) was determined for each feature. The closer a feature is to this maximum point, the more stably it performs. Multiple features can share the same rank.

Some studies also perform boxplot analysis to characterize radiomic feature stability and/or reproducibility [31,34]. To compare the results from the stability score with the feature’s boxplot, we also performed a boxplot analysis for one feature as an example, namely Skewness.

In addition, the analysis for First Order features was repeated for benign and malignant lesions separately (Figures S4–S7 in the Supplementary Materials).

### 2.6. Statistical Analysis

A Shapiro–Wilk test was used to test the feature value Skewness for normal distribution. In the case of normal distribution, a two-tailed Student’s test was used to test for significance. In case of non-normal distribution, the paired Wilcoxon rank sum test was used. A *p*-value below 0.05 was considered to be significant. Bonferroni correction was applied to account for multiple testing.

To assess the uncertainty, 95% confidence intervals (CIs) were calculated for the stability metrics. Specifically, the CIs for the OCCC were determined using 1000 bootstrap iterations. In contrast, CIs for the DR were derived using the Matlab statistical package. Shape features were excluded from the shift evaluations because there is no effect on these features when applying shifts. By analyzing the plot of CIs, the statistical dependence of different features can be assessed based on the amount of CI overlap.

## 3. Results

In total, *n* = 125 patients with *n* = 195 lesions were initially considered for evaluation. In the initial visual image quality check, *n* = 44 lesions were excluded from the study due to image distortions (*n* = 29 lesions), insufficient image quality (*n* = 12 lesions), lesion distorted by biopsy (2 lesions), or because the lesion was not visible in any image contrast including DCE (1 lesion). Two lesions were excluded as the MR images were taken after the patient had undergone surgery (one lesion) or patient information was incomplete (one lesion). In total, 103 patients aged between 24.1 and 86.4 years (48.8 years [median], 49.7 [mean], 12.6 [standard deviation]) with 149 lesions were used for further evaluation.

A total of 70 lesions (46.98%) were histologically categorized as malignant (amongst invasive lobular carcinoma (ILC), invasive carcinoma of no special type (NST), mamma carcinoma without further details (Maca), clear cell breast cancer (CCBC), ductal carcinoma in situ (DCIS)), and 79 lesions (53.02%) were classified as benign (amongst cysts, fibroadenoma, and mastopathy). A follow-up result was available for all but two benign lesions. These two lesions without follow-up examination were defined as simple cysts in sonography.

The range, mean, median, and standard deviation of the maximum lesion diameter for benign lesions were [4.10; 32.81] mm, 8.73 mm, 6.84 mm, and 4.99 mm. For malignant lesions, these metrics were the following: [5.47; 41.02] mm, 11.97 mm, 10.39 mm, and 6.37 mm. Table S2 in the Supplementary Materials lists the segmentation size values for all thresholds (th = 80%, th = 90%, th = 100%, th = 110%, and th = 120%). The smallest segmentation amongst all used thresholds was 2.73 mm for th = 120% (see image of a benign lesion (cyst) in Figure S8 in the Supplementary Materials). Additionally, Figures S9 and S10 show the distribution of dice coefficients and percentage size change for the different thresholds.

**Figure 2:**
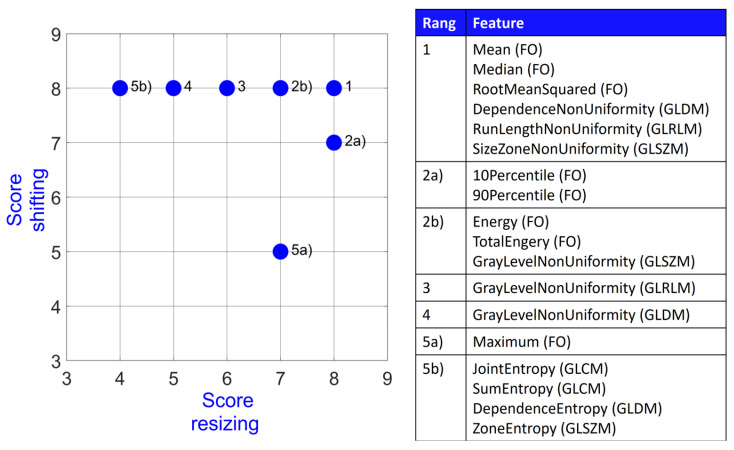
Stability score for 18 most stable features amongst all feature classes.

Figure 2 visualizes the stability score for the 18 most stable features out of all feature classes but Shape (FO, GLCM, GLRLM, GLDM, GLSZM, and NGTDM). Overall, these include eight First Order features, two GLCM features, two GLRLM features, three GLDM features, three GLSZM features, and no NGTDM features. The best performing features are the three First Order features Mean, Median, and RootMeanSquared together with the GLDM feature DependenceNonUniformity, the GLRLM feature RunLengthNonUniformity, and the GLSZM feature SizeZoneNonUniformity. All of these 18 features had at least a score of 5 (OCCC points + DR points) for shifting and score of 4 (OCCC points + DR points) for resizing.

**Figure 3:**
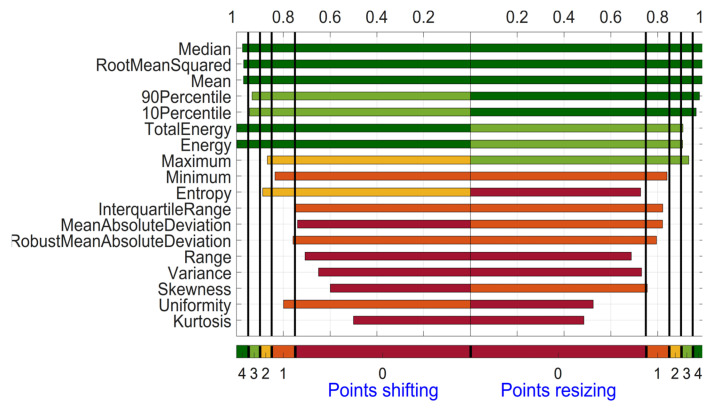
Overall Concordance Correlation Coefficient for First Order features derived from the ADC maps. Features are sorted based on the summed OCCC score for both categories from top to bottom.

As an example, Figure 3 shows the OCCCs for the 18 First Order features. For the category shifting, 5 out of these 18 features (Median, RootMeanSquared, Mean, TotalEnergy, and Energy) scored 4 points for OCCC, having the largest (most stable) OCCC approximately equal to 1.00 and Kurtosis having the smallest (most unstable) OCCC with 0.50 in the category shifting. For the category resizing, 5 out of these 18 features (Median, RootMeanSquared, Mean, 90Percentile, and 10Percentile) scored 4 points for OCCC. Median and Mean have the most stable OCCC with 0.99, and Kurtosis again has the smallest (most unstable) OCCC with 0.48. The largest difference of OCCCs for one feature between the two categories is found for Uniformity with 0.28 (OCCC = 0.80 for shifting and OCCC = 0.52 for resizing). Most similar are the OCCCs of the two categories for the feature Minimum (OCCC = 0.84 for shifting and OCCC = 0.84 for resizing).

**Figure 4:**
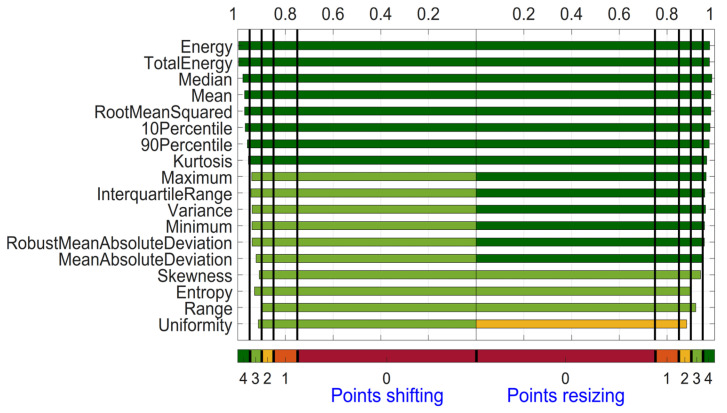
Dynamic Range for First Order features derived from ADC map. Features are sorted based on the summed OCCC score for both categories from top to bottom.

Figure 4 shows the DRs for both categories for the 18 First Order features. For the category shifting, 8 out of the 18 features scored 4 points (Energy, TotalEnergy, Median, Mean, RootMeanSquared, 10Percentile, 90Percentile, and Kurtosis). Energy and TotalEnergy have the highest (most stable) DR value rounded at 1.00 and Range the smallest (most unstable) DR with 0.90. For the category resizing, all 18 features but 4 (Skewness, Entropy, Range, and Uniformity) scored 4 points. Median performs best with DR = 0.99, and Uniformity has the smallest (most unstable) DR with 0.88. The largest difference in DR for a feature between the two categories is found for Skewness with 0.03 (DR = 0.91 for shifting and DR = 0.94 for resizing). Most similar are the DR values of the two categories for the feature Median (DR = 0.98 for shifting and DR = 0.98 for resizing).

**Figure 5:**
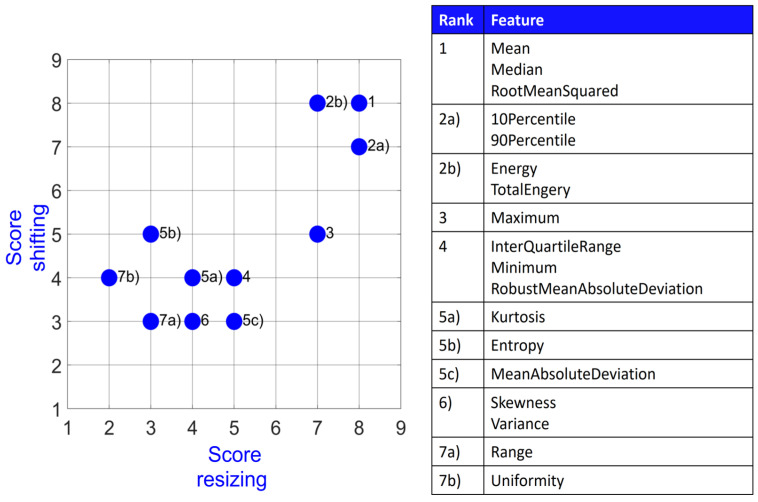
Stability score for First Order features.

Figure 5 depicts the final stability score for the 18 First Order features graphically for both shifting and resizing. Every feature had at least a score of 3 in shifting and a score of 2 in resizing. Mean, Median, and RootMeanSquared have the highest possible score of 8 for both categories. Range and Uniformity share the last rank, whereas Range has a score of 3 in both categories and Uniformity performs better in shifting (score 4) compared to resizing (score 2).

**Figure 6:**
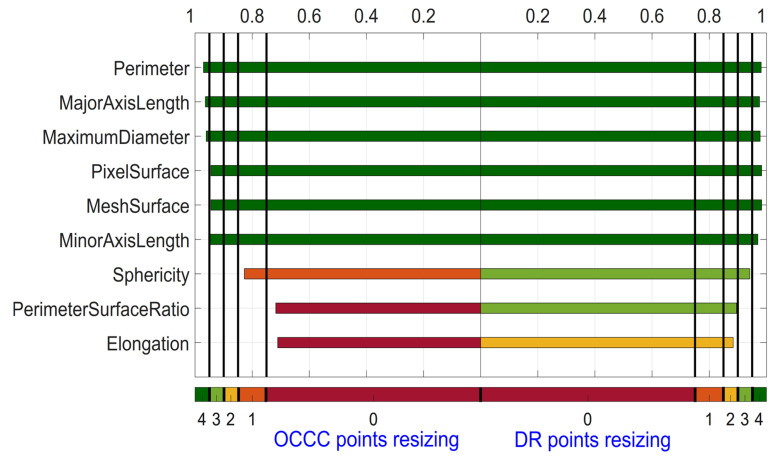
OCCC and Dynamic Range for Shape features derived from ADC map for the category variation of threshold. Features are sorted based on the summed OCCC and DR score from top to bottom.

Figure 6 depicts the results for OCCC and DR for the nine Shape features, which was based solely on the varied Otsu’s threshold. Perimeter, MajorAxisLength, MaximumDiameter, PixelSurface, MeshSurface and MinorAxisLength have 4 points for both OCCC and DR analysis. Perimeter has the highest OCCC with 0.97, and Elongation has the lowest with OCCC = 0.71. The highest DR is found for Perimeter, MajorAxisLength, MaximumDiameter, PixelSurface, and MeshSurface with 0.98. The lowest DR is found for Elongation with DR = 0.88.

**Figure 7:**
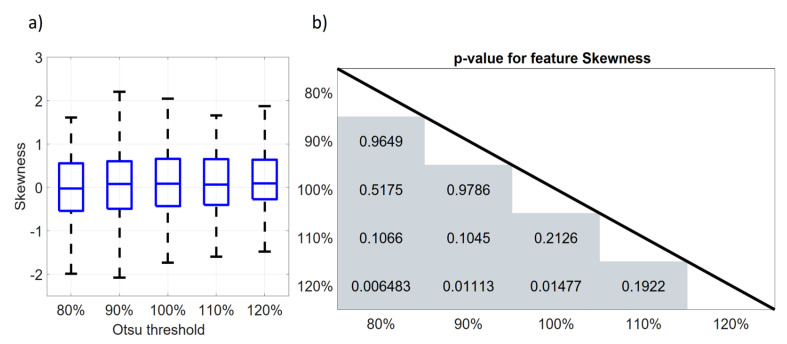
Boxplots and significance values for First Order feature Skewness for resizing. (**a**) Boxplot, (**b**) matrix with *p*-values from paired Wilcoxon rank sum test.

To compare our approach to test for feature robustness with the boxplot analysis performed in former studies, Figure 7a shows a boxplot for the First Order feature Skewness for the five different thresholds used for segmentation. It is clearly visible that the feature does not show significant differences between the thresholds. As the feature Skewness was not normally distributed, a paired Shapiro–Wilk test with Bonferroni correction was used to test for significant differences between the thresholds. The corresponding *p*-values from the Shapiro–Wilk test are displayed in Figure 7b. There is no significant difference (*p* > 0.05/10) in Skewness for all studied threshold pairs.

**Figure 8:**
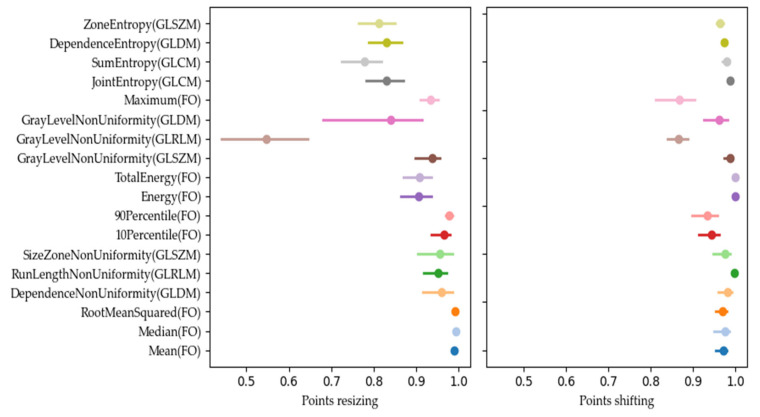
OCCC scores, marked with a dot, and their confidence interval (CI), marked with a line, for the 18 most stable features for resizing (**left**) and shifting (**right**).

Our analysis of the CIs of the OCCCs and DRs revealed different widths of the CIs for the 18 stable radiomic features. Many features had overlapping CIs, as shown in Figure 8, where the OCCC CIs after resizing overlapped for JointEntropy (GLCM), SumEntropy (GLCM), DependenceEntropy (GLDM), and ZoneEntropy (GLSZM), for example.

**Figure 9:**
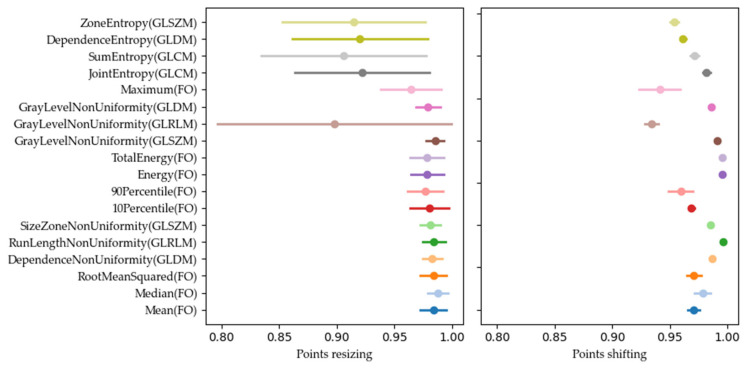
DR scores, marked with a dot, and their confidence interval (CI), marked with a, for the 18 most stable features for resizing (**left**) and shifting (**right**).

In addition, some features, such as GrayLevelNonUniformity (GLRLM) for DR values after resizing (Figure 9), revealed wider CIs, indicating higher variability and lower stability. These features may be less reliable for consistent use in clinical settings.

## 4. Discussion

In this study, radiomic features extracted from diffusion-weighted images of breast lesions were evaluated with regards to their stability against modifications of the position and shape of the segmentation masks. The results demonstrate that radiomic features derived from DWI seem to have varying degrees of intrinsic robustness against modifications of the segmentation masks and that this stability is further dependent on the type of modification of the segmentation.

In this study, two different types of variations of the segmentations were used to assess the stability of features. First, segmentations were shifted by one pixel to the left, right, up, and down, thus no change in shape was induced (shifting). Second, the shape of the segmentations was varied by changing the Otsu’s threshold that the segmentation was built on (resizing). The types of variation were chosen based on the translational clinical consideration of varying characteristics of segmentations: Shape-wise very similar segmentations in between readers and/or timepoints that are, however, shifted slightly in their location or varying segmentation approaches including either larger or smaller proportions of a lesion. The variations further reflect the current literature in context of radiomic feature stability tests. Bologna et al. tested for feature stability in soft tissue sarcomas in DWI by applying different geometric variations to the segmentations and comparing the extracted features afterwards [10]. This procedure is comparable to the shift of segmentations performed in our analysis. Liu et al. performed a stability analysis of radiomic features with respect to segmentation variation in oropharyngeal cancer in CT imaging [35]. They rescaled the original segmentation by shrinking and magnifying, which is comparable to the increase and decrease in the Otsu’s threshold in this paper.

The stability of features is an important aspect when it comes to the translation of advanced image analysis techniques in oncologic imaging aiming to provide additive insights for lesion characterization or stratifying treatment pathways. DWI herein is of special interest due to its capability of deriving different oncologically relevant microstructural correlates from the acquisitions [17,36,37]. A high stability of the features thus might indicate an increasing translational relevance for the usage of DWI radiomics studies in breast lesions.

Zhang et al. quantified the robustness of features extracted from diffusion-weighted images of breast lesions, among others, against delineation differences using the intra-class correlation coefficient (ICC) [38]. They performed erosion and dilation of the segmentations to generate multiple diverse segmentations per lesion. They reported the ICCs for breast DWI features against all variations of segmentation to be distributed in a wide range. The greatest effect on feature values and thus the largest ICC range was obtained by the maximum dilatation performed. They presented the number of robust features (ICC  ≥  0.9) identified from the diverse segmentations graphically in the form of a bar plot. As they did not report the names of the robust features but rather concentrated on the number of robust features amongst the different variations, we cannot compare if our robust features align with their results. Spick et al. tested DWI as a radiomic imaging bio-marker in breast lesions by investigating the reproducibility, repeatability, and diagnostic accuracy of mean ADC [39]. As they did not test other features besides the mean ADC, a comprehensive comparison to our study is difficult. The feature stability study from Grazier et al. uses different MR acquisitions, but besides that, very similar results to the present one can be seen [40]. They evaluated the robustness of MR radiomics features with respect to variations in the manual tumor segmentation of T1-weighted breast cancer images. They used two different software programs for feature extraction, one being the same as in our study (Pyradiomics). Our findings that Mean, Median, and RootMeanSquared are stable First Order features and RunLengthNonUniformity is a stable GLRLM feature are in line with Grazier et al., who reported an intra-class correlation coefficient (ICC) > 0.9 for these features. In our study, DependenceNonUniformity from the GLDM class and SizeZoneNonUniformity from the GLSZM class were also ranked as most robust. According to Granzier et al., these features have an ICC < 0.9, whereby DependenceNonUniformity is the 4th out of 14 most stable ones in the GLDM class with an ICC close to 0.9 (ICCs were only graphically presented in [40]) and SizeZoneNonUniformity was reported as rank 9 out of 16 GLSZM features with an ICC also close to 0.9 [40]. A comparison of the shape-based features was only possible to a limited extent, as Granzier et al. worked with 3D segmentations and thus the extracted Shape features differ from the ones in the current study for 2D segmentations. The most stable Shape feature for the current study was Perimeter, which is not calculated for 3D segmentations. The same holds true for PixelSurface and MeshSurface, which are both ranked as stable in the present study but were not analyzed in [40], whereby the 3D version of PixelSurface, namely VoxelVolume, was indeed ranked as stable. MajorAxisLength, MaximumDiameter, and MinorAxisLength were calculated but not listed amongst the most stable features by Granzier et al. [40]. 

Our findings regarding stable radiomic features in breast DWI can be considered in line with the reported ones for alternating MR acquisitions using T1-weighted breast MR imaging sequences when concentrating on features other than Shape-based ones.

As already mentioned in the introduction, Peerlings et al. evaluated the stability of radiomic features in DWI by means of a test–retest study for different organs and filed strengths [11]. The features extracted from colorectal liver metastases may be compared best to the features in the current study, as the images were also taken in 3 T scanners, but from different vendors (Siemens Magnetom Trio Tim, Siemens Healthineers AG, Erlangen, Germany; Philips Ingenia, Philips, Amsterdam, The Netherlands; GE Discovery 750 w, GE HealthCare, Chicago, IL, USA). The Concordance Correlation Coefficient (CCC) was calculated to assess feature stability. Features with CCC > 0.85 were classified as stable. Besides the two First Order features Mean and RootMeanSquared and the GLSZM feature SizeZoneNonUniformity, the rest of the six most stable features (excluding Shape features) from the current study were also assigned as robust by Peerlings et al. (Median (FO), DependenceNonUniformity (GLDM), and RunLengthNonUniformity (GLRLM)) [11]. These three features were not only classified as stable, but they were also amongst the most stable ones in their respective feature class. As Peerling et al. used 3D segmentations, only the Shape features MajorAxisLengths, MaximumDiameter, and MinorAxisLength could be compared regarding the most stable Shape features in this study. All three were classified as stable with a CCC > 0.85. They used a different tool for feature extraction, but all discussed features were defined exactly as the features from this study. With three exceptions and the fact that not all Shape features could be compared, the results from the current study can thus be seen as in line with results from liver DWI.

Radiomics stability was studied as well in T1-weighted and T2-weighted images by Jensen et al. in healthy liver parenchyma using Pyradiomics [31]. Our findings that Mean, Median, and RootMeanSquared are stable First Order features when it comes to variations in segmentation are in line with their results. DependenceNonUniformity (GLDM), RunLengthNonUniformity (GLRLM), and SizeZoneNonUniformity (GLSZM), on the other hand, were assigned a low stability by Jensen et al. [31]. They did not consider Shape features, so no comparison can be made here.

Radiomic features’ stability in Turbo-Spin-Echo phantom images across different MR systems with different field strengths by investigating the intrascanner Coefficient of Variation (COV) and the interscanner ICC was investigated by Rai et al. [41]. Again, the three features Mean, Median, and RootMeanSquared were ranked as the best performing features in the First Order class. Similar alignment was found for RunLengthNonUniformity (GLRLM) and SizeZoneNonUniformity (GLSZM), which were both claimed to be one of the best performing features in their respective feature class. No comparison for DependenceNonUniformity from the GLDM class could be made, as this feature class was not included by Rai et al. [41]. None of the shape features that were ranked stable in this study were ranked stable in Rai et al., either as the feature was not extracted or it did not fulfil the stability criteria (COV < 20% and ICCC > 0.8).

Roy et al. investigated radiomic feature stability in T1- and T2-weighted MR images of breast cancer tumors with respect to noise, resolution, and tumor volume [42]. They found that the investigated First Order features (excluding Skewness and Kurtosis) are less sensitive to changes in the signal-to-noise ratio (SNR) than features of other classes. This finding might be a possible explanation for the high stability found in this feature class in this study, where 8 out of the 18 most stable features were First Order features. GLRLM and GLSZM were the classes most sensitive to changes in SNR [42].

Given these findings of previous studies, the First Order features that were ranked stable in our study also seem to be stable in other body regions, across different scanners, and when extracted from MR images with other contrasts than those obtained in DWI. Regarding the other most stable features of our study, DependenceNonUniformity (GLDM), RunLengthNonUniformity (GLRLM), and SizeZoneNonUniformity (GLSZM), results on their stability in the literature are not as consistent, however. Comparing Shape-based features in terms of stability is difficult as extracted Shape features vary amongst different studies or are not extracted at all.

It is remarkable that the First Order feature Skewness did not show a significant difference between any segmentation pair of investigated thresholds nor in the boxplot representation of Figure 7, but it did only reach a score of 4 out of 8 in resizing (OCCC: 1 point, DR: 3 points) and a score of 3 out of 8 in shifting (OCCC: 0 points, DR: 3 points). This suggests that the pairwise comparison of features from different segmentations should not be used solely to investigate feature robustness. If only the difference in features’ median values is taken into account, features with a wide range but close to zero median might be mistakenly classified as robust based on the stable median. This could be prevented by using the OCCC and DR which also measure the dispersion of the feature values. It thus seems recommendable not to use only boxplot analyses to address feature stability but to additionally include more comprehensive analyses like, e.g., those in our study or the study by Jensen et al. [31].

### Limitations

Our study has some limitations. First, feature stability was assessed by comparing feature values extracted from slightly different segmentations. However, image pre-processing, feature extraction setup, and test–retest analysis were not taken into account when ranking features. As these points are challenging in radiomic studies, further investigations are needed.

Second, the stability of Shape features could only be based on the analysis of the segmentations gained by changing the Otsu’s threshold, and shifting the segmentation was not considered. The stability analysis of the other feature classes was therefore more comprehensive.

Third, our analysis was conducted using MRI scanners from a single vendor at a single institution, specifically 3T MRI scanners. This represents an unavoidable limitation in terms of the generalizability of our findings. The features extracted and the results obtained in our study were not tested for stability across different scanners, field strengths, acquisition times, or protocol variations. As a result, results may vary when using equipment from other manufacturers, different field strengths, or different sequence characteristics. This limitation is particularly relevant because variations in scanner technology, field strength, and imaging protocols may affect the quality and characteristics of the acquired images, potentially affecting the reproducibility and robustness of our findings. Future research should aim to address this limitation by conducting multi-institutional studies that include a variety of MRI scanners from different manufacturers and field strengths. In addition, examining the stability and consistency of imaging characteristics across different acquisition parameters and time points will be critical to improving the generalizability of radiomic analyses.

Fourth, stability analysis was based on 2D ROIs in this paper. It would be interesting to repeat the analysis for 3D ROIs and compare the results. This includes as well that due to the retrospective character of the data analysis, it was not possible to additively investigate a test–retest scenario that considers potential additive bias due to acquisition variations. Fifth, for two lesions, the b50 and b750 images had to be registered due to non-negligible patient motion. The registration might have an effect on the extracted features. As only two lesions were affected, no statistically valid analysis could be performed on this sub-dataset, but it seems unlikely that a large effect on the overall feature stability would be present. In addition, we deemed it more holistic to determine feature stability independently of lesion type. Thus, feature stability was analyzed based on both benign and malignant lesions. A separate analysis divided into benign and malignant lesions can be found in the Supplementary Materials (See Figures S4–S7). The analysis yields essentially similar results. Importantly, one should be aware that the variation in the segmentation can influence the resulting feature stability. Especially adapting Otsu’s threshold can have a huge effect on the extracted features. The used thresholds were therefore selected in such a way that they overlap with the original segmentation (th = 100%) in an acceptable range.

Further, the described stability of the features depends on the thresholds of the underlying stability score thresholds. While we chose the thresholds based on established practices in the literature, different thresholds might have altered the ranking of the features.

Lastly, the study focused only on feature stability and did not consider the discriminative ability to distinguish between malignant and benign lesions. Although the most stable features do not overlap with the most important features according to Zhang et al., which may lead to a loss of information, focusing on stable features improves reproducibility and consistency [43]. Future research should concentrate on the clinical effect of using only stable features.

## 5. Conclusions

In conclusion, the radiomics features Mean (FO), Median (FO), RootMeanSquared (FO), DependenceNonUniformity (GLDM), RunLengthNonUniformity (GLRLM), SizeZoneNonUniformity (GLSZM), Perimeter (Shape), MajorAxisLength (Shape), MaximumDiameter (Shape), PixelSurface (Shape), MeshSurface (Shape), and MinorAxisLength (Shape) were found to be the most robust across segmentation variations in diffusion-weighted images of breast lesions. The significantly varying stability of different radiomics features with some features being influenced even by slightly altered segmentations needs to be considered when performing radiomics studies and in discussing the translational potential of advanced image analysis techniques.

## Figures and Tables

**Table 1:** Imaging protocol.

Parameter	DWI Sequence	DCE Sequence
Sequence	Single-shot echo planar	T1 Dixon
Orientation	Transverse	Transverse
Repetition time (ms)	6290–8695	5.41–5.97
Echo time (ms)	66	Echo time 1, TE1: 2.46, Echo time 2, TE2: 3.69
Voxel size (mm^3^)	2.7 × 2.7 × 4 with in plane interpolation to 1.35 × 1.35 × 4	0.8 × 0.8 × 1.5
Fat suppression	Inversion recovery Inversion time TI = 220, 250 ms for the Skyra Inversion time TI = 250 ms for the Vida	-
Field of view (mm^2^)	350 × 218	360 × 360 for the Skyra, 380 × 380 for the Vida
Matrix	128 × 80	448 × 448
Slice thickness (mm)	4	1.5
No. of slices	39 to 45	112
Multi-slice mode	Interleaved	Sequential
Parallel imaging	GeneRalized Autocalibrating Partial Parallel Acquisition (GRAPPA)	GeneRalized Autocalibrating Partial Parallel Acquisition (GRAPPA)
Acceleration factor	2	3
Bandwidth (Hz/pixel)	2300 for the Skyra, 2298 for the Vida	800 for the Skyra 860 for the Vida
Acquisition time (min)	3:34 to 4:56	6:21 to 7:24
b-values (s/mm^2^)	50, 750, 1500	-
Averages	3, 8, 15, or 20	-
Diffusion	3D diagonal, i.e., along the direction (1, 1, 1) in the scanner coordinate system; single-refocused with vendor-provided eddy current correction ‘dynamic field correction’	-

**Table 2:** Stability score.

Points	OCCC	DR
0	<0.75	<0.75
1	[0.75, 0.85[	[0.75, 0.85[
2	[0.85, 0.9[	[0.85, 0.9[
3	[0.9, 0.95[	[0.9, 0.95[
4	≥0.95	≥0.95

## Data Availability

The datasets generated and analyzed during the current study are not publicly available due to general data protection regulation, GDPR, but are available from the corresponding author upon reasonable request.

## Supplementary Materials

The following supporting information can be downloaded at https://www.mdpi.com/article/10.3390/diagnostics14131427/s1, Table S1: Settings used for feature extraction; Figure S1: Example image matrix together with GLCM, GLRLM, GLDM, and GLSZM matrices for feature calculation. Different colors are used to track the different steps; Figure S2: Example image matrix together with NGTDM procedure. Different colors are used to track the different steps; Figure S3: Resulting NGTDM matrix for procedure shown in Figure S5; Figure S4: Stability score for all feature classes but Shape based on benign lesions exclusively; Figure S5: Stability score for all feature classes but Shape based on malignant lesions exclusively; Figure S6: OCCC and DR for shape features based on benign lesions exclusively; Figure S7: OCCC and DR for shape features based on malignant lesion exclusively; Table S2. Segmentation size values (range, mean, median, and standard deviation) for all used threshold values (th = 80%, th = 90%, th = 100%, th = 110%, and th = 120%); Figure S8: b1500 image of benign lesion with the smallest segmentation (shown in green) in this study. The segmentation was based on th = 120%; Figure S9: Dice coefficients for different thresholds; Figure S10: Size ranges for different thresholds.

## References

[1] Freitas A.J.A., Causin R.L., Varuzza M.B., Calfa S., Hidalgo Filho C.M.T., Komoto T.T., Souza C.D.P., Marques M.M.C. (2022). Liquid Biopsy as a Tool for the Diagnosis, Treatment, and Monitoring of Breast Cancer. Int. J. Mol. Sci..

[2] Yeo S.K., Guan J.L. (2017). Breast Cancer: Multiple Subtypes within a Tumor?. Trends Cancer.

[3] Roulot A., Héquet D., Guinebretière J.-M., Vincent-Salomon A., Lerebours F., Dubot C., Rouzier R. (2016). Tumoral heterogeneity of breast cancer. Ann. Biol. Clin..

[4] Nakaura T., Higaki T., Awai K., Ikeda O., Yamashita Y. (2020). A primer for understanding radiology articles about machine learning and deep learning. Diagn. Interv. Imaging.

[5] Bodalal Z., Trebeschi S., Nguyen-Kim T.D.L., Schats W., Beets-Tan R. (2019). Radiogenomics: Bridging imaging and genomics. Abdom. Radiol..

[6] Lambin P., Leijenaar R.T.H., Deist T.M., Peerlings J., de Jong E.E.C., van Timmeren J., Sanduleanu S., Larue R.T.H.M., Even A.J.G., Jochems A. (2017). Radiomics: The bridge between medical imaging and personalized medicine. Nat. Rev. Clin. Oncol..

[7] Conti A., Duggento A., Indovina I., Guerrisi M., Toschi N. (2021). Radiomics in breast cancer classification and prediction. Semin. Cancer Biol..

[8] Liu Z., Li Z., Qu J., Zhang R., Zhou X., Li L., Sun K., Tang Z., Jiang H., Li H. (2019). Radiomics of Multiparametric MRI for Pretreatment Prediction of Pathologic Complete Response to Neoadjuvant Chemotherapy in Breast Cancer: A Multicenter Study. Clin. Cancer Res..

[9] Satake H., Ishigaki S., Ito R., Naganawa S. (2022). Radiomics in breast MRI: Current progress toward clinical application in the era of artificial intelligence. Radiol. Med..

[10] Bologna M., Montin E., Corino V.D., Mainardi L.T. Stability assessment of first order statistics features computed on ADC maps in soft-tissue sarcoma. Proceedings of the 39th Annual International Conference of the IEEE Engineering in Medicine and Biology Society (EMBC).

[11] Peerlings J., Woodruff H.C., Winfield J.M., Ibrahim A., Van Beers B.E., Heerschap A., Jackson A., Wildberger J.E., Mottaghy F.M., DeSouza N.M. (2019). Stability of radiomics features in apparent diffusion coefficient maps from a multi-centre test-retest trial. Sci. Rep..

[12] Ramli Z., Karim M.K.A., Effendy N., Rahman M.A.A., Kechik M.M.A., Ibahim M.J., Haniff N.S.M. (2022). Stability and Reproducibility of Radiomic Features Based on Various Segmentation Techniques on Cervical Cancer DWI-MRI. Diagnostics.

[13] Jones D.K. (2011). Diffusion MRI: Theory, Methods, and Applications.

[14] Partridge S.C., Nissan N., Rahbar H., Kitsch A.E., Sigmund E.E. (2017). Diffusion-weighted breast MRI: Clinical applications and emerging techniques. J. Magn. Reson. Imaging.

[15] Iima M., Partridge S., Le Bihan D. (2022). Diffusion MRI of the Breast.

[16] Bogner W., Gruber S., Pinker K., Grabner G., Stadlbauer A., Weber M., Moser E., Helbich T.H., Trattnig S. (2009). Diffusion-weighted MR for differentiation of breast lesions at 3.0 T: How does selection of diffusion protocols affect diagnosis?. Radiology.

[17] Bickelhaupt S., Steudle F., Paech D., Mlynarska A., Kuder T.A., Lederer W., Daniel H., Freitag M., Delorme S., Schlemmer H.P. (2017). On a fractional order calculus model in diffusion weighted breast imaging to differentiate between malignant and benign breast lesions detected on X-ray screening mammography. PLoS ONE.

[18] Pistel M., Laun F.B., Bickelhaupt S., Dada A., Weiland E., Niederdränk T., Uder M., Janka R., Wenkel E., Ohlmeyer S. (2022). Differentiating Benign and Malignant Breast Lesions in Diffusion Kurtosis MRI: Does the Averaging Procedure Matter?. J. Magn. Reson. Imaging.

[19] Partridge S.C., Amornsiripanitch N. (2017). DWI in the Assessment of Breast Lesions. Top. Magn. Reson. Imaging.

[20] Palm T., Wenkel E., Ohlmeyer S., Janka R., Uder M., Weiland E., Bickelhaupt S., Ladd M.E., Zaitsev M., Hensel B. (2019). Diffusion kurtosis imaging does not improve differentiation performance of breast lesions in a short clinical protocol. Magn. Reson. Imaging.

[21] Schneider C.A., Rasband W.S., Eliceiri K.W. (2012). NIH Image to ImageJ: 25 years of image analysis. Nat. Methods.

[22] Otsu N. (1975). A threshold selection method from gray-level histograms. Automatica.

[23] Bickelhaupt S., Jaeger P.F., Laun F.B., Lederer W., Daniel H., Kuder T.A., Wuesthof L., Paech D., Bonekamp D., Radbruch A. (2018). Radiomics Based on Adapted Diffusion Kurtosis Imaging Helps to Clarify Most Mammographic Findings Suspicious for Cancer. Radiology.

[24] Wagner F., Laun F.B., Kuder T.A., Mlynarska A., Maier F., Faust J., Demberg K., Lindemann L., Rivkin B., Nagel A.M. (2017). Temperature and concentration calibration of aqueous polyvinylpyrrolidone (PVP) solutions for isotropic diffusion MRI phantoms. PLoS ONE.

[25] Van Griethuysen J.J., Fedorov A., Parmar C., Hosny A., Aucoin N., Narayan V., Beets-Tan R.G., Fillion-Robin J.C., Pieper S., Aerts H.J. (2017). Computational Radiomics System to Decode the Radiographic Phenotype. Cancer Res..

[26] Zwanenburg A., Vallières M., Abdalah M.A., Aerts H.J.W.L., Andrearczyk V., Apte A., Ashrafinia S., Bakas S., Beukinga R.J., Boellaard R. (2020). The Image Biomarker Standardization Initiative: Standardized Quantitative Radiomics for High-Throughput Image-based Phenotyping. Radiology.

[27] Lawrence I., Lin K. (1989). A concordance correlation coefficient to evaluate reproducibility. Biometrics.

[28] Barnhart H.X., Haber M., Song J. (2002). Overall concordance correlation coefficient for evaluating agreement among multiple observers. Biometrics.

[29] Stevenson M. (2015). epiR: Tools for the Analysis of Epidemiological Data. https://CRAN.R-project.org/package=epiR.

[30] Balagurunathan Y., Gu Y., Wang H., Kumar V., Grove O., Hawkins S., Kim J., Goldgof D.B., Hall L.O., Gatenby R.A. (2014). Reproducibility and Prognosis of Quantitative Features Extracted from CT Images. Transl. Oncol..

[31] Jensen L.J., Kim D., Elgeti T., Steffen I.G., Hamm B., Nagel S.N. (2021). Stability of Liver Radiomics across Different 3D ROI Sizes-An MRI In Vivo Study. Tomography.

[32] Baeßler B., Weiss K., Dos Santos D.P. (2019). Robustness and Reproducibility of Radiomics in Magnetic Resonance Imaging: A Phantom Study. Investig. Radiol..

[33] Balagurunathan Y., Kumar V., Gu Y., Kim J., Wang H., Liu Y., Goldgof D.B., Hall L.O., Korn R., Zhao B. (2014). Test-retest reproducibility analysis of lung CT image features. J. Digit. Imaging.

[34] Wang H., Zhou Y., Wang X., Zhang Y., Ma C., Liu B., Kong Q., Yue N., Xu Z., Nie K. (2021). Reproducibility and Repeatability of CBCT-Derived Radiomics Features. Front. Oncol..

[35] Liu R., Elhalawani H., Mohamed A.S.R., Elgohari B., Court L., Zhu H., Fuller C.D. (2020). Stability analysis of CT radiomic features with respect to segmentation variation in oropharyngeal cancer. Clin. Transl. Radiat. Oncol..

[36] Someya Y., Iima M., Imai H., Yoshizawa A., Kataoka M., Isoda H., Le Bihan D., Nakamoto Y. (2022). Investigation of breast cancer microstructure and microvasculature from time-dependent DWI and CEST in correlation with histological biomarkers. Sci. Rep..

[37] Iima M., Yano K., Kataoka M., Umehana M., Murata K., Kanao S., Togashi K., Le Bihan D. (2015). Quantitative non-Gaussian diffusion and intravoxel incoherent motion magnetic resonance imaging: Differentiation of malignant and benign breast lesions. Investig. Radiol..

[38] Zhang X., Zhong L., Zhang B., Zhang L., Du H., Lu L., Zhang S., Yang W., Feng Q. (2019). The effects of volume of interest delineation on MRI-based radiomics analysis: Evaluation with two disease groups. Cancer Imaging.

[39] Spick C., Bickel H., Pinker K., Bernathova M., Kapetas P., Woitek R., Clauser P., Polanec S.H., Rudas M., Bartsch R. (2016). Diffusion-weighted MRI of breast lesions: A prospective clinical investigation of the quantitative imaging biomarker characteristics of reproducibility, repeatability, and diagnostic accuracy. NMR Biomed..

[40] Granzier R.W.Y., Verbakel N.M.H., Ibrahim A., van Timmeren J.E., van Nijnatten T.J.A., Leijenaar R.T.H., Lobbes M.B.I., Smidt M.L., Woodruff H.C. (2020). MRI-based radiomics in breast cancer: Feature robustness with respect to inter-observer segmentation variability. Sci. Rep..

[41] Rai R., Holloway L.C., Brink C., Field M., Christiansen R.L., Sun Y., Barton M.B., Liney G.P. (2020). Multicenter evaluation of MRI-based radiomic features: A phantom study. Med. Phys..

[42] Roy S., Whitehead T.D., Quirk J.D., Salter A., Ademuyiwa F.O., Li S., An H., Shoghi K.I. (2020). Optimal co-clinical radiomics: Sensitivity of radiomic features to tumour volume, image noise and resolution in co-clinical T1-weighted and T2-weighted magnetic resonance imaging. EBioMedicine.

[43] Zhang Q., Peng Y., Liu W., Bai J., Zheng J., Yang X., Zhou L. (2020). Radiomics Based on Multimodal MRI for the Differential Diagnosis of Benign and Malignant Breast Lesions. J. Magn. Reson. Imaging.

