# Supplementary material for: Stability of Radiomic Features against Variations in Lesion Segmentations Computed on Apparent Diffusion Coefficient Maps of Breast Lesions
Source: Diagnostics (Basel). 2024 Jul 3;14(13):1427. doi: 10.3390/diagnostics14131427 (PMC11241112; doi:10.3390/diagnostics14131427)
Supplement: Supplementary file 1 [file diagnostics-14-01427-s001.zip › diagnostics-2995287-supplementary.pdf]

*Supplementary Materials*

# Stability of radiomic features computed on ADC maps of breast lesions

**Table S1:** Settings used for feature extraction.

| **Setting** | |
|---|---|
| imageType: | Original |
| featureClass: | firstorder<br>shape<br>glcm<br>glrlm<br>glszm<br>gldm<br>ngtdm |
| setting: | binCount: 50<br>voxelArrayShift: 0<br>force2D: true<br>resegmentRange: [0, 3.5] |

**Extracted features**

**First Order features:** Describe distribution of voxel intensities within the segmentation through commonly used and basic metrics. Extracted from original image.

**Shape features:** Describe size and shape of the segmentation independent of voxel intensities within the segmentation. Extracted from original image.

**GLCM features:** Gray Level Co-occurrence Matrix (GLCM) describes distribution of a specific pixel combination i,j in a specific direction and at a defined distance. (i, j)th element of GLCM represents the number of times the combination of the intensity values i and j occurs in two pixels of the image matrix that have a distance $\delta$ at an angle $\theta$ to each other. Distance $\delta$ from the central pixel is defined by the infinity norm. In 2D, a central pixel for $\delta=1$ has exactly 8 neighbouring pixels. Figure S4 shows a two-dimensional example of an image matrix and the corresponding GLCM for $\delta=1$ (pixels exactly one pixel apart) and $\theta=0$ (pixels to the left and right of the central pixel).

**GLRLM features:** Gray Level Run Length Matrix (GLRLM) quantifies grey value distribution of the pixels in the image matrix. (i, j)th element of the GLRLM describes how often a certain grey level run of length j and intensity i occurs along the angle $\theta$. Figure S4 shows an example of how a GLRLM for $\theta=0$ can be obtained from the image matrix.

**GLDM features:** Gray Level Dependence Matrix (GLDM) describes gray level dependencies in the image matrix. A grey level dependency is defined as the number of connected pixels at a distance $\delta$, which are dependent on the central pixel. The pixel j is dependent on the central pixel i, if $|i - j| \le \alpha$ holds for a defined $\alpha$. The (i, t)th element of the GLDM indicates the number of times in the image

matrix a pixel with grey value i is combined with j dependent neighbouring pixels. For $\alpha$=0 and δ=1 the GLDM for a two-dimensional image matrix is calculated (see Figure S4).

**GLSZM features:** Gray Level Size Zone Matirx (GLSZM) describes grey value zones in the image matrix. A grey level zone is defined as the number of connected pixels with the same grey level. Two pixels count as connected if they have a distance of 1 under the infinity norm (δ=1). (i, j)th element of the GLSZM specifies how often the image matrix contains the grey value zone with value i and size j. Unlike the GLCM and the GLRLM, the GLSZM is determined for all angles in a matrix. Figure S4 shows an exemplary image matrix with calculated GLSZM.

**NGTDM features:** Neighbouring Gray Tone Difference Matrix (NGTDM) describes the difference between a grey value and the average grey value of the neighbouring pixels at a distance δ. Finally, the sum of the absolute grey value differences is included in the matrix. In Figure S5, the sum of the absolute grey value differences for the respective pixel intensities i (here 1-4) is calculated for a 2D matrix. In the resulting NGTDM matrix, the respective grey value i is listed in the first column. The second column indicates how often the respective grey value occurs in the image matrix. The probability of the respective grey value in relation to the entire image matrix is contained in the third column. The last column contains the calculated sums of the absolute grey value differences.

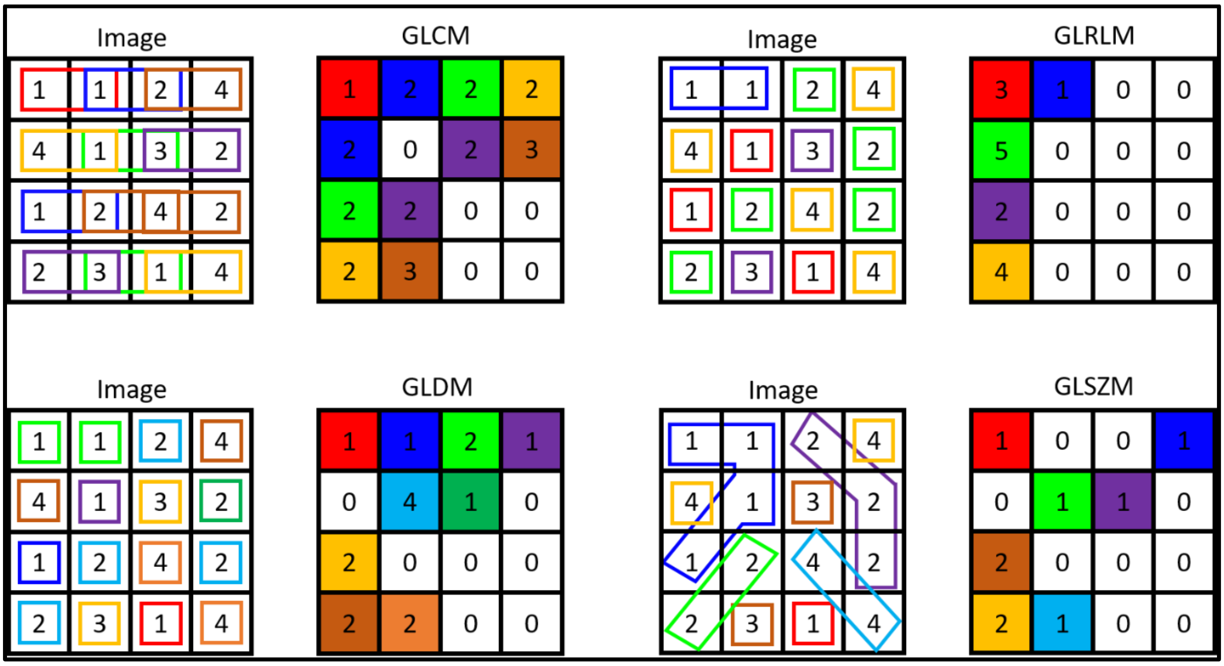


**Figure S1:** Example image matrix together with GLCM, GLRLM, GLDM, and GLSZM Matrices for Feature calculation. Different colors were used to track the different steps.

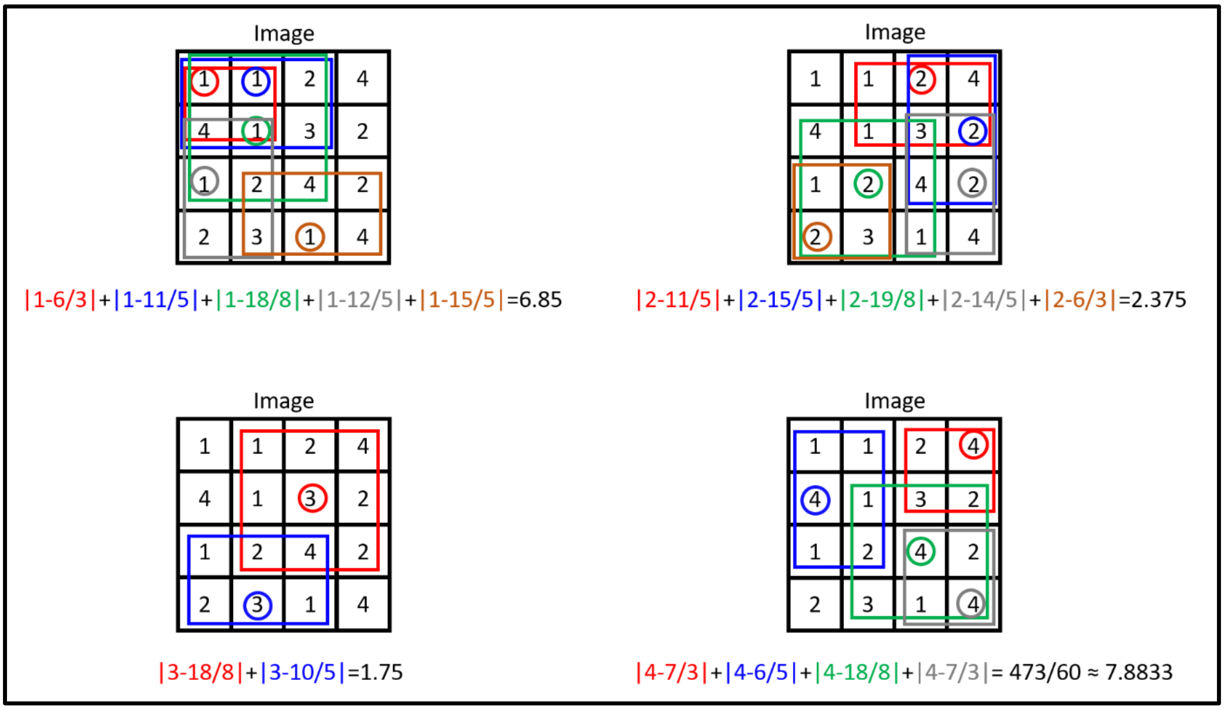


**Figure S2:** Example image matrix together with NGTDM procedure. Different colors were used to track the different steps.

| | | | |
|---|---|---|---|
| 1 | 5 | 0.3125 | 6.85 |
| 2 | 5 | 0.3125 | 2.375 |
| 3 | 2 | 0.125 | 1.75 |
| 4 | 4 | 0.25 | 7.8833 |

**Figure S3:** Resulting NGTDM Matrix for procedure shown in Figure S2.

**Feature analysis divided in benign and malignant lesions:**

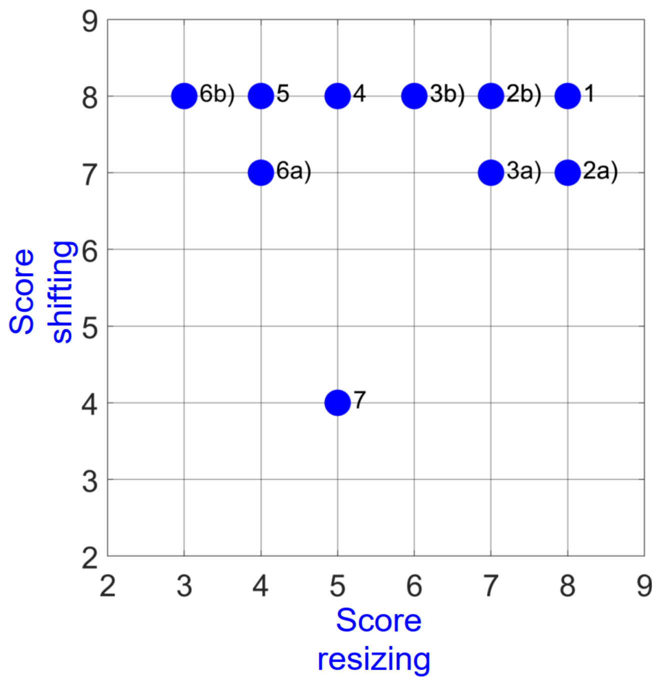


| Rank | Feature |
|---|---|
| 1 | Mean (FO)<br>Median (FO)<br>RootMeanSquared (FO) |
| 2a) | 90Percentile (FO)<br>Maximum (FO) |
| 2b) | DependenceNonUniformity (GLDM)<br>RunLengthNonUniformity (GLRLM)<br>SizeZoneNonUniformity (GLSZM) |
| 3a) | 10Percentile (FO) |
| 3b) | Energy (FO)<br>TotalEngery (FO)<br>GrayLevelNonUniformity (GLSZM) |
| 4 | GrayLevelNonUniformity (GLRLM) |
| 5 | JointEntropy (GLCM)<br>DependenceEntropy (GLDM) |
| 6a) | GrayLevelNonUniformity (GLDM) |
| 6b) | ZoneEntropy (GLSZM) |
| 7 | RobustMeanAbsoluteDeviation (FO) |

**Figure S4:** Stability score for all feature classes but Shape based on benign lesions exclusively.

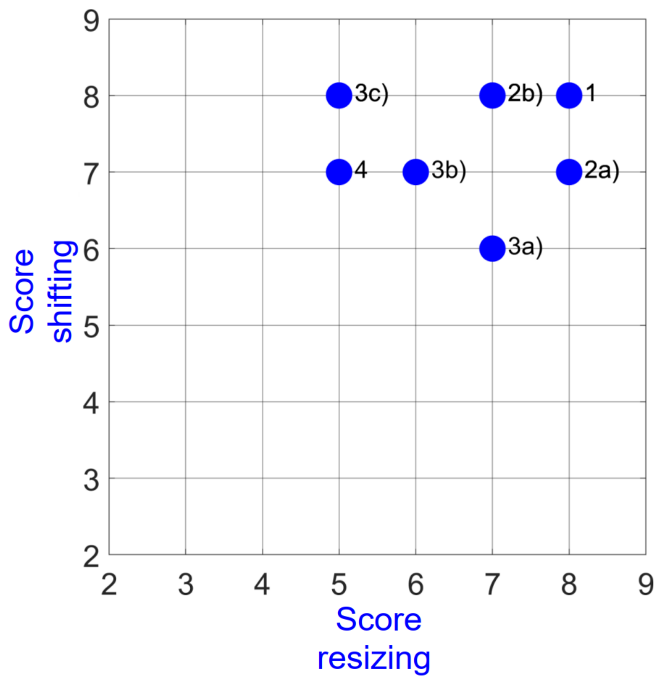


| Rank | Feature |
|---|---|
| 1 | Median (FO)<br>DependenceNonUniformity (GLDM)<br>RunLengthNonUniformity (GLRLM)<br>GrayLevelNonUniformity (GLSZM)<br>SizeZoneNonUniformity (GLSZM) |
| 2a) | 10Percentile (FO)<br>Mean (FO)<br>RootMeanSquared (FO) |
| 2b) | Energy (FO)<br>TotalEngery (FO)<br>GrayLevelNonUniformity (GLDM)<br>GrayLevelNonUniformity (GLRLM) |
| 3a) | Minimum (FO) |
| 3b) | Imc2 (GLCM) |
| 3c) | JointEntropy (GLCM)<br>DependenceEntropy (GLDM)<br>ZoneEntropy (GLSZM) |
| 4 | Imc1 (GLCM) |

**Figure S5:** Stability score for all feature classes but Shape based on malignant lesions exclusively.

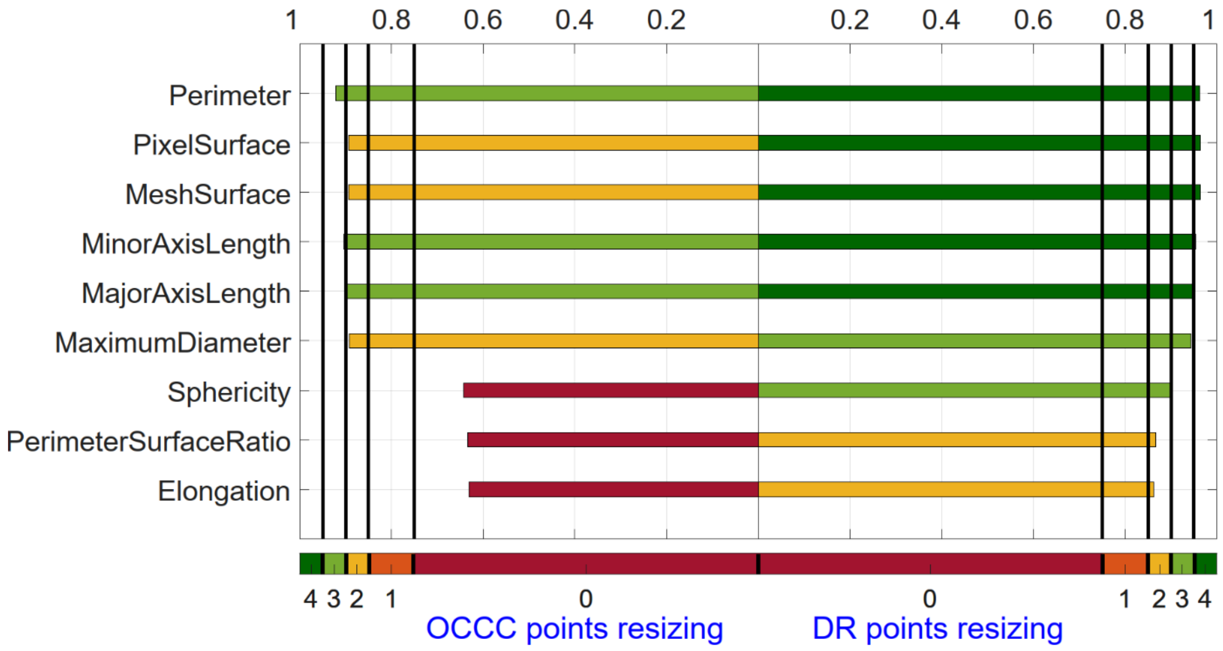


**Figure S6:** OCCC and DR for shape features based on benign lesions exclusively.

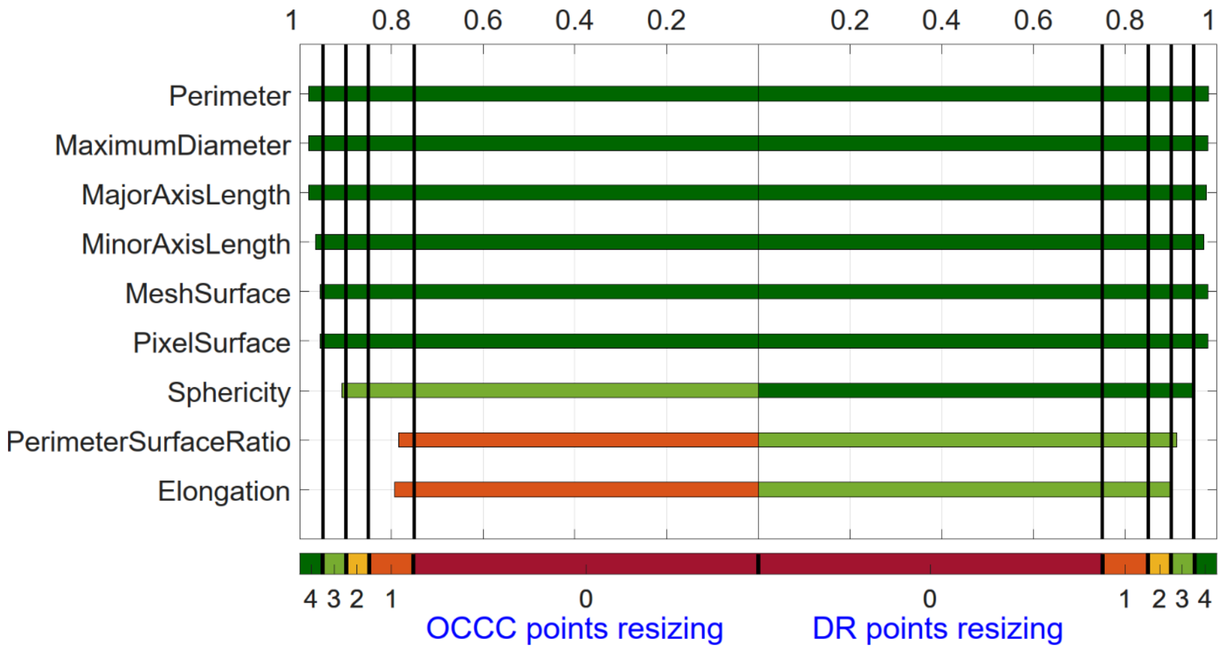


**Figure S7**: OCCC and DR for shape features based on malignant lesion exclusively.

**Table S2:** Segmentation size values (range, mean, median, standard deviation) for all used threshold values (th=80%, th=90%, th=100%, th=110%, th=120%).

| | | Benign | Malignant |
|---|---|---|---|
| Range, Mean, Median, standard deviation in mm | th=80% | [5.47; 34.18] (10.60, 9.57, 5.37) | [5.47; 49.22] (13.69, 11.78, 7.17) |
| | th=90 % | [4.10; 32.81] (9.50, 8.20, 5.09) | [5.47; 43.75] (12.93, 10.94, 6.60) |
| | th=100% | [4.10; 32.81] (8.73, 6.84, 4.99) | [5.47; 41.02] (11.97, 10.39, 6.37) |
| | th=110% | [4.10; 31.45] (7.96, 6.84, 4.61) | [4.10; 39.65] (11.40, 9.71, 6.19) |
| | th=120% | [2.73; 25.98] (6.98, 5.47, 3.93) | [4.10; 36.91] (10.41, 8.44, 5.59) |

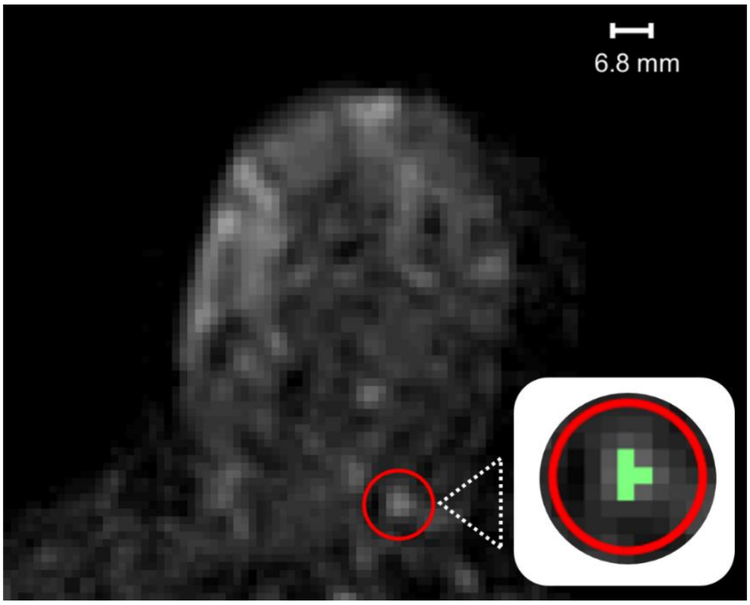


**Figure S8:** b1500 image of benign lesion with the smallest segmentation (shown in green) in this study. The segmentation was based on th=120%.

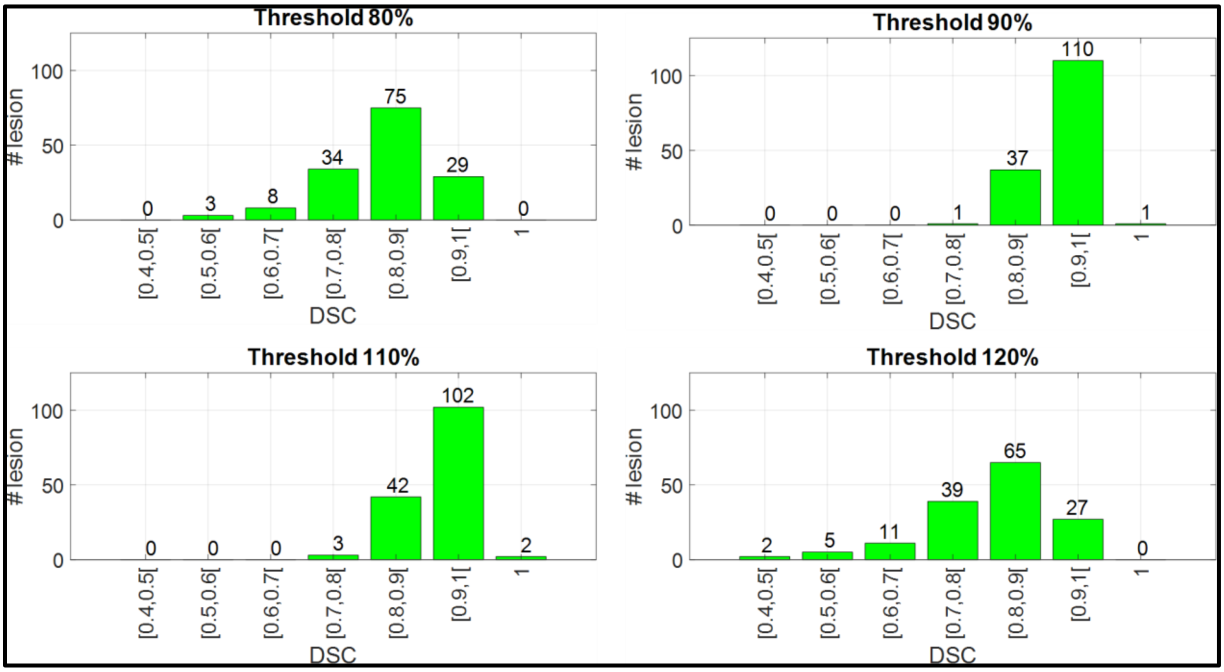


**Figure S9:** Dice coefficients for different thresholds.

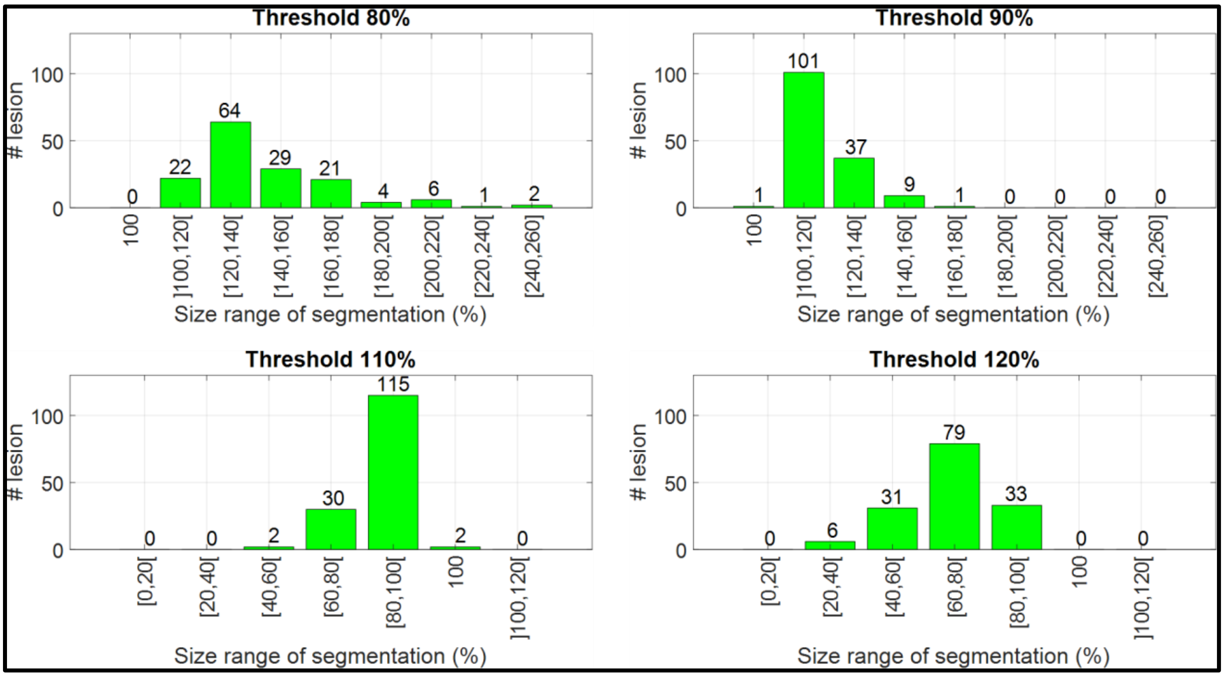


**Figure S10:** Size ranges for different thresholds.
